# Allocation of Land Factors in China Looking Forward to 2035: Planning and Market

**DOI:** 10.3390/ijerph20043424

**Published:** 2023-02-15

**Authors:** Yuzhe Wu, Jia Ao, Yuhang Ren

**Affiliations:** 1 School of Public Affairs, Zhejiang University, Hangzhou 310058, China; 2 Center for Balance Architecture, Zhejiang University, Hangzhou 310028, China

**Keywords:** territorial space planning, land market, factors allocation, production–living–ecological spaces, modernization

## Abstract

Land factors are natural resources with fundamental and strategic significance in the achievement of China’s 2035 modernization goals. Dilemmas caused by market-oriented or planning-oriented allocation of land factors urgently call for new theoretical guidance and mode. After conducting a systematic review of the literature, this paper built a new framework from the perspective of production–living–ecological spaces to facilitate a better understanding of China’s land factors allocation looking forward to 2035. Inductive and deductive methods were both used to interpret the applications of planning and market in land factors allocation. Our results show that: (1) The allocation of land factors for production space is truth-oriented and needs the guidance of market efficiency. The essential feature of “production” as the driving force in production space requires that the allocation of land factors in production space must “respect rules, give play to the agglomeration effect, and rationally carry out regional economic layout”. (2) For the allocation of land factors for living space, it is necessary to pursue a kindness-oriented approach and establish a reasonable housing supply system based on people. Among them, the ordinary commercial housing and improving housing should rely on market forces to achieve multi-subject supply, while affordable housing should be ensured through government intervention in a multi-channel way. (3) For the allocation of land factors in ecological space, aesthetic-oriented planning should follow the rule of territorial differentiation and realize the transformation of ecological function into ecological value through market mechanisms. Top-down planning and bottom-up market represents the logic of overall and individual rationality, respectively. The effective allocation of land factors requires the utilization of both planning and market forces. However, the intersection needs be guided by boundary selection theory. This research indicates that “middle-around” theory could be a possible theoretical solution for future study.

## 1. Introduction

Modernizing China’s governance system for territorial space and capacity is an important part of China’s efforts to achieve the strategic goal of socialist modernization by 2035 [1]. Looking backward, China’s rapid development in the past 40 years was mainly driven by the factors represented by land. In particular, the successful reform of land factors from total planning allocation to market allocation supported the rise of China’s industrial manufacturing industry, the improvement of people’s living conditions, the large-scale construction of urban infrastructure, and the accumulation of social wealth [2]. However, since Rittle and Webber introduced the concept of planning as a thorny problem in the 1970s, today, in the 21st century, scholars are still baffled by the problem of planning for complex urban environments [3]. The core of the complexity of planning comes from human behavior, whether it is the behavior of supply producers or consumers, which will often form feedback loops. Good planning is, first of all, rational and should conform to the balance of market supply and demand [4].

As a traditional agricultural country, China is trying to embrace industrial civilization in the process of becoming a modern country. However, facing the basic national conditions of more people with less land, China’s modernization process must achieve high-quality development of production, satisfy people’s demand for a better life and at the same time protect the fragile ecological environment to ensure the sustainability of modern development, which requires China’s modern development to be guided by ecological civilization. In this regard, China’s central government has put forward the overall requirements for land and space development of “intensive and efficient production space, livable and moderate living space, and beautiful ecological space”. However, China’s relatively lagging territorial space governance system and reform capacity do not adapt to the huge changes surrounding land factors. In the process of rapid urbanization and industrialization in China, many contradictions have arisen due to the uncoordinated planning and market means in the allocation of land factors. Disordered competition and interest conflicts among local governments and departments have led to extensive land use. The existence of the dual structure of urban and rural land has led to the dilemma that the urbanized agricultural population is facing insufficient urban housing security and difficulties in realizing rural land property rights. The regional division of development strategy lacks the support of a flexible planning mechanism for the allocation of land factors, resulting in a lack of necessary incentives and funds for ecological and environmental protection, which makes it difficult to truly achieve the goal of ecological civilization construction.

In 2019, China announced the establishment of a new spatial planning system, which integrates the traditional functional zoning planning, land use planning, and urban and rural planning [5]. This new system provides basic principles for various development and protection activities and provides a sustainable spatial development guide for realizing the strategic goal of national modernization by 2035. However, the realization of these grand development strategies requires the removal of the contradictions accumulated in the previous development stage. Central to this problem is to straighten out the relationship between planning and market in the allocation of land factors [6]. Existing theoretical research either focuses on the guiding role of government planning [7,8], or focuses on the regulating role of market allocation [9], or emphasizes that both are indispensable [10], which does not reveal enough about the boundary selection theory between the two. Therefore, this paper aims to address the achievement of effective allocation of land factors looking toward 2035 by exploring a new framework and theoretical guidance.

## 2. Literature Review

### 2.1. The Allocation of Land Factors as Quasi-Public Goods

Public goods refer to the products and services consumed by the whole society, such as national defense, public transportation, urban disaster prevention facilities, etc. Public goods are characterized by high input, low return, and social necessities [11,12]. According to the different degree of competition and exclusivity, public goods can be divided into pure public goods and quasi-public goods. Generally, it is the responsibility of governments to provide purely public goods such as defense and security. Quasi-public goods, however, can be provided in a more flexible and diversified way according to their different degrees of publicity [13]. Land resources have typical characteristics of quasi-public goods, which need market and planning allocation in the real world. On the one hand, land is an important factor of production, and market mechanism is a panacea to achieve the sustainability of land supply [14]. Gebre and Demsis [15] surveyed public-private partnerships in Ethiopia’s road sector, citing the lack of government funding, the inability of the public sector to shoulder all project risks, social pressures on people due to poor road infrastructure, the need for private sector skills and experience, and the need to improve service levels as the main reasons for their cooperation. The results of the study provide solutions to problems related to the delivery of road infrastructure [15]. On the other hand, land resources are quasi-public goods, whose allocation needs government intervention. This is because weak competition environment (Cournot competition) can bring more public benefits than strong competition environment (Bertrand competition) [16]. For instance, Rohman evaluated the role of the government in the public-private partnership toll road project in Indonesia [17]. In the consideration of land public goods, some governments gradually attract the private sector to provide infrastructure through public-private partnership. In addition, land use has externalities. For the negative externalities of environmental pollution, Li Sufeng et al. [18] built a dynamic game model and analyzed the evolution and development law of carbon emission reduction between governments and enterprises. It is believed that carbon trading, as one of the effective market tools combining planning and market, can promote the smooth implementation of the “dual carbon” goal [18].

### 2.2. Planning Allocation of Land Factors

Keynesianism believes that due to market failure, the government must actively intervene to correct the defects of the market mechanism, and planning is one of the most important and effective ways for the government to intervene in the allocation of land resources [19]. Although many countries began to carry out territorial planning in succession from the early 20th century, most western countries did not begin to take effective steps to regulate and intervene in regional development until after the Second World War. Since the 1960s, due to the rapid industrial development and accelerated urbanization in Western countries, the problems of population, resources and environment have become prominent, especially the imbalance of regional development has become increasingly serious. Among them, the development problem of backward areas is very prominent, while some prosperous core areas, such as Paris in France, the United Kingdom and the southeast area centered around London, appear the problem of excessive concentration. in order to overcome the phenomenon of “over-density” (over-concentration) and “over-sparse” (low level of development) of the national industry in the region and realize the balanced development of national economy, France [20], Britain [21] and other developed countries have respectively adopted relevant policies to strengthen the planning and guidance of regional development. Even in the United States, which has the most developed market economy, planning as a public policy plays an important role in resource allocation [22].

From the general experience of developed countries in the world, space planning, as an important public policy, is an important means for the government to conduct space governance. The International Habitat Conference II has set “adequate housing for all” as the core goal of the Habitat Agenda [23], and the right to housing is listed as a part of the “right to a minimum standard of living” in the Universal Declaration of Human Rights [24]. Therefore, planning should first meet peoples’ housing needs. Later, the New Urban Agenda of Habitat III re-established the core status of cities in the world human settlement environment, and pointed out that the planning should focus on the whole urban system rather than a single urban element, that is to say, not only the planning of a single element such as the road network structure, but also the solution of many complex problems [25]. These complex issues include resilient cities, housing issues, food issues, etc. For example, Sadegh Sabouri et al. explored the national practice of linking and coordinating transportation and land use planning in the United States. The ultimate goals of these projects were found to be similar throughout the case studies, namely to reduce suburban sprawl and the associated need for road construction, and to create more livable, sustainable, walkable, cyclable and passable communities within the region [26]. When it comes to China, in the deep development period of urbanization from “extension type” to “concursive type”, spatial planning focuses on solving urban life problems such as dislocation of employment and housing, traffic congestion, environmental pollution, insufficient supporting public facilities and unbalanced development with surrounding areas [27]. In addition, government planning and allocation of land is a multi-objective public management activity, which can be in line with the 2030 Sustainable Development Goals (SDGs). In the 2030 Development Agenda, 17 Sustainable Development Goals and 169 targets are divided into three dimensions, namely “economic development”, “environmental well-being”, and “social inclusion”. Domestic and foreign scholars have studied the integration strategies of urban planning and resource management from the perspectives of social stability and development as well as ecological environmental protection [28,29,30,31,32,33].

### 2.3. Market Allocation of Land Factors

The neoliberalism represented by Hayek criticized the government failure of Keynesian intervention. Hayek believed that the “invisible hand” of the market was highly capable of self-regulation and risk prevention in complex market economic activities [34]. Since the late 20th century, the global society has been influenced by the neoliberal theory, which has also penetrated the land factors allocation. The marketization mechanism of land resource allocation is to follow the rule of value, the law of competition, the law of supply and demand and other market laws to spontaneously reach the optimal allocation state. Under the condition of market mechanism, land rights holders will utilize land resources in accordance with the principle of profit maximization and promote intensive land use. Whether any land is developed for commercial, residential or public services, market needs, and development interests will usually give the best choice [35]. In developed capitalist economies, it can be seen that there is a “market” model represented by the United States, in which the allocation of housing resources largely depends on the incentive of information channels and price mechanisms [36]. Deegen and Halbritter [37] analyzed the problem of pure land allocation under certain market conditions when land use changes have different impacts on commodity prices and production factor prices, and proposed three different models: a completely open economy, a closed economy and an economy in which selection prices are determined externally [37]. China, with its socialist market economy system, is no exception. The transformation of urban land from planned allocation to market-oriented allocation is an important part of China’s market-oriented economic reform, and the marketization of land transfer can significantly promote economic growth in the long run [38].

## 3. Allocation of Land Factors in China: Modes and Dilemmas

### 3.1. Planning-Oriented Allocation Mode and Its Performance

Planning is future-oriented, statutory, and holistic. As a government action, it can make up for the failure and absence of market allocation and correct the disadvantages of market allocation such as external diseconomy and information asymmetry. As a resource in the planning system, the allocation of land factors is regarded as a part of China’s macro-control, and it is also regarded as a policy tool of state governance. In the allocation of land factors, planning mainly plays a leading and controlling role. Since China’s reform and opening-up in 1978, the central government has given full play to the leading and controlling role of planning in the allocation of land factors through the preparation of land use planning. The law guarantees the effective implementation of the plan. In the compilation of China’s first round of overall land use planning (Outline of the National Land Use Planning (1986–2000)), indicators and zoning are two policy tools that constitute the overall land use plan. Specifically, it first analyzes and evaluates the utilization suitability of all land factors, and then it divides the available land factors into cultivated land indicators and construction land indicators. After that, it allocates them among different regions, departments, and industries, to ensure land factors better serve the various needs of national economic development. However, on the one hand, the lack of science and public participation in the overall land use plan compiled, and the persistent pursuit of the perfection of the planning results lead to a disconnect between the overall land use plan and the actual needs of social and economic development. On the other hand, under the impact of China’s market economy reform, the lack of constraints and flexibility of land use planning have led to a serious loss of cultivated land resources in China. Therefore, in the preparation of the second round of overall land use planning (Outline of the National Land Use Planning (1997–2010)), the protection of valuable cultivated land resources has become the primary goal. The cultivated land protection indicators approved by the central government are allocated from top to bottom among local governments at different levels, to realize the guidance and containment of development demand from supply side of land factors. However, in practice, the relationship between the protection pressure of cultivated land and the expansion of construction land demand has not been effectively coordinated, which has challenged the forward-looking planning. In the preparation of the third round of overall land use planning (Outline of the National Land Use Planning (2006–2020)), the practice of local governments on the relationship between protection and development in land use provided valuable information for the preparation of overall land use planning. In this stage, land use control has become the most distinctive feature of China’s land use planning system. This system has played an important role in ensuring the realization of multi-dimensional goals of ecological environment protection, dynamic balance of cultivated land resources, and intensive use of construction land.

### 3.2. Market-Oriented Allocation Mode and Its Performance

As a resource in the market system, market allocation tools should play a fundamental and decisive role in land factors. Land is the spatial carrier for human economic and social activities and the basic resource to be used. The increasingly diversified demands for land resources from population growth, urban expansion, and social development objectively require the market mechanism to play its role of allocating scarce land resources. From a micro perspective, the market promotes the effective conversion of land resources between different uses through the price mechanism, competition mechanism, and supply and demand mechanism, and maximizes the use value of land resources. From a macro perspective, as a factor commodity in national economic and social development, the improvement of its overall allocation efficiency still requires clear property rights and reduced market transaction costs as a prerequisite [39]. As the reform and opening up, the market allocation of rural land has greatly contributed to the increase of agricultural production and provided capital and labor accumulation for industrialization and urbanization. The marketization of urban land lease has significantly contributed to China’s economic growth in the long term through two major channels: the financing effect and the resource allocation effect. The construction of industrial parks and real estate development in the context of urban land market allocation has led to the rapid development of industrialization and urbanization in China. The activation of the capital properties of land has given rise to the land finance driven urban development and management mode, which on the one hand has accumulated wealth for urban development, but on the other hand, the drawbacks of the land finance mode have become increasingly evident [40].

### 3.3. Dilemmas from the Unclear Boundary between Planning and Market Allocation

In general, the planning role of land factor allocation emphasizes “top-down”, which has the disadvantage of over-idealized planning allocation and often generates unavoidable conflicts between the rigidity of planning and the uncertainty and complexity of reality [3]. When planning allocation is dominant, distortion and misallocation of land factors arises. In addition, institutional factors, such as the lack of public participation and supervision, may make it difficult for the planning to allocate land factors as expected. It is foreseeable that the increasingly improved territorial spatial planning system will overcome the drawbacks of “multiple planning”, promote the role of planning tools in land factors allocation, and promote the modernization of territorial spatial governance system and governance capacity, but this requires a more flexible, effective, and adequate interface between planning intervention and market adjustment [41]. In contrast, the market role of land factor allocation emphasizes “bottom-up”. However, market allocation tends to focus on short-term interests and there are external diseconomies in the market mechanism, which leads to disorder and imbalance when market allocation is dominant, manifesting in extensive use of industrial land, duplication of construction and overcapacity, high housing prices and inadequate housing security, local debt and land financial risks, and environmental pollution problems.

The effective combination of market mechanism and government planning is an indispensable basis for realizing the Pareto optimization of land factors allocation. With the increasing complexity and suddenness of human economic and social development, the effective integration of the two becomes more important than ever. However, in the current allocation of land factors, the boundary between planning and market is not clear, which has created hurdles for their effective integration. Therefore, how to draw the boundary between the two is the most significant issue.

## 4. Allocation of Land Factors from the Perspective of Production-Living-Ecological Spaces

**Figure 1:**
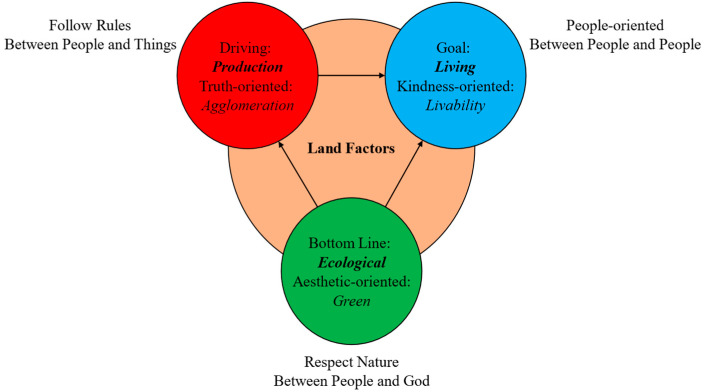
Allocation of land factors from the perspective of production-living-ecological spaces.

Production–living–ecological spaces is a classification perspective of land factors allocation (Figure 1). Firstly, Production is a driving force. Production space is truth-oriented, a philosophy between people and things, which follows rules and emphasizes agglomeration effect. Secondly, Living is goal. Living space is kindness-oriented, a philosophy between people and people, which follows the logic of people-oriented and emphasizes livability. Thirdly, Ecology is the bottom line. Ecological space is aesthetic-oriented, a philosophy between people and God, which needs to respect nature and stresses the guidance of green development. Based on the above reasons, this study will explore the synergistic application of planning and market in land factors allocation from the perspective of production–living–ecological spaces.

### 4.1. Allocation of Land Factors for Production Space

The market-based reform of land allocation can unleash huge potential for economic development and is of great significance to achieving steady and high-quality economic growth. The land factor allocation oriented by market efficiency is firstly reflected in the agglomeration of population and various production factors in geographical space, and behind this agglomeration of factors is the agglomeration of industries. Take Dongguan City in Guangdong province as an example. In 2020, the total population of the city reached 10.47 million, and the global market share of the computer mouse, keyboards and capacitors had reached 70%. For this concentration of market allocation, Adam Smith opened the Wealth of Nations with an example of making paper clips: The maximum efficiency of a single person making paper clips is 1 to 20 per day (depending on their proficiency), while a production chain of 10 people can produce 48,000 per day, an average of 4800 per person, with an efficiency increase of 240 to 4800 times. The improvement of efficiency is the result of the division of production, which is precisely based on the premise of agglomeration. In other words, the possibility of division of labor can only be provided by clustering to a certain extent. In addition, if enterprises in a certain industry cluster in a region, it will also attract enterprises from other industries to engage in production and operation in the region [42]. At this time, the agglomeration economy broke through industrial boundaries and was called the “urbanization economy”. From international experience, the size of a town is closely related to economic development. According to the World Bank report, more than half of the people in the high-income countries category live in big cities with populations of more than 1 million, and less than a quarter live in small towns with populations of less than 20,000. The opposite is true in low-income countries, where only about 1 in 10 people live in large cities with populations of more than 1 million and nearly three-quarters live in small towns with populations of less than 20,000 (Table 1). The agglomeration rule is universal, including China. Therefore, the allocation of land factors in production space should follow the law and emphasize the agglomeration effect, and the development of big cities should become the support for the construction of small cities.

From the above perspective, population agglomeration brings the scale effect of production exchange and promotes the development of urban innovation, production, and trade. However, on the negative side, the population agglomeration leads to pollution, crowding and other problems. When the positive externalities brought by the urban agglomeration effect cannot make up for the negative impacts brought by the urban problems, the social relations within the city will deteriorate. Therefore, while giving full play to the decisive role of the market in factors allocation, it is more important to rationally plan the regional economic layout, and earnestly do a good job in basic security work such as territorial and spatial planning, land property rights system and land legal system.

### 4.2. Allocation of Land Factors for Living Space

The living space is people oriented. The allocation of land factors needs planning and market to both guide a reasonable housing supply system. As mentioned in the report, Chinese modernization is characterized by a huge population, common prosperity for all the people, harmony between material and spiritual civilization, harmonious coexistence between man and nature, and the path of peaceful development. So, in this context, how to build China’s housing system for 2035?

First, in the real estate market, we should focus on absorbing the rigid demand caused by the current population structure and improving the demand. In the face of such a huge population in China, it is impossible to rely on the government alone, so we need to rely on the market power and multi-subject supply. The housing demand for ordinary commodity housing can be realized through the distribution function of the free market, whose production, distribution, circulation, and consumption are regulated by the market mechanism. The operation of the real estate market with fierce competition and its supply structure (such as high-grade commodity apartments, villas, etc.) are basically within the scope of complete marketization. Besides maintaining the rules of fair competition, the government mainly decides the relationship between supply and demand according to market rules and market mechanism. Only in this way can we effectively increase supply, meet diversified demand, and improve the efficiency of housing resource allocation. Although the demand for ordinary commodity housing can be realized through the distribution function of the free market, ordinary commodity housing still needs to adhere to the “housing does not stir” requirement. A profit tax could be one option to curb property speculation. In addition, with the commercialization and liberalization of housing, the rate of home ownership has been greatly increased, and people have a higher pursuit of material and spiritual, and the demand for improving housing has become the main body of the real estate market, among which the ecological housing that realizes the harmonious modernization of human and nature can also be regarded as a kind of improving housing. Although there are no complete statistics, it is predicted in the relevant research report that there are many vacant houses, and many families own multiple homes. The “heavy transaction, light possession” housing tax system has no longer meet the needs of the current economic and social development. Levying resource occupancy tax on housing has become an important policy choice and the source of new land transfer fees in the future. Levying taxes and fees on the link of housing ownership and appropriately reducing the transaction tax burden can revitalize the stock of real estate, reduce the vacancy rate, and make full use of land and housing resources.

Second, under the market economy system, in order to realize the modernization of common prosperity for all the people and ensure that everyone has a house to live in, the government needs to implement some special policies and measures to help the floating population groups separating from their household registrations to solve the housing difficulties. The general term for this policy system is called the housing security system. However, the unbalanced development of housing security in the new era is mainly manifested in three aspects: the unbalanced distribution of supply and demand between cities, the unbalanced distribution of urban interior space, and the unbalanced construction and management of affordable housing. The construction of affordable housing ignores the internal demand difference between big cities and small and medium-sized cities, leading to short supply in big cities and oversupply in small and medium-sized cities. Public housing and low-rent housing are usually built far away from industrial urban centers, with poor transportation infrastructure, high commuting costs, and inadequate public services. Qualification audit and follow-up supervision of affordable housing are not in place. In view of the unbalanced development of housing security, the author believes that under the background of new urbanization, it is necessary to carry out planning allocation of land factors, formulate housing policies, and promote urban-rural integration. Through the planning and promotion of affordable housing, rail transit terminals in big cities should become an important choice of affordable housing. At the same time, only when the rural floating population has housing security in the city can the reform of rural homestead system be leveraged in a real sense to improve farmers’ property income and achieve common prosperity.

### 4.3. Allocation of Land Factors for Ecological Space

During the 14th Five-Year Plan period, China’s ecological civilization construction entered a critical period with carbon reduction as the key strategic direction, promoting the synergistic effect of carbon reduction and pollution reduction, promoting the comprehensive green transformation of economic and social development, and realizing the improvement of ecological environment quality from quantitative to qualitative change. Ecological space is guided by beauty, and the external requirements of transforming carbon to achieve carbon neutrality and peak carbon dioxide emissions are the internal driving force of ecological civilization construction. At present, the shortage of available land in China is becoming increasingly serious, while the demand for land use is constantly increasing, which requires us to effectively carry out territorial space planning and ecological environmental protection, by drawing various security bottom lines, implementing use control and ecological restoration.

First, the ecological pattern still needs to be planned according to the rules of territorial differentiation. Among them, the ecological protection red line is the lifeline to ensure ecological security and an important means for the state to control ecological space. It is necessary to use scientific methods to identify high-value carbon sink spaces, quantitatively assess the functional value of forest, grassland, wetland, ocean, and other ecological systems protection in carbon sinks, and incorporate their spatial location into the ecological protection red line of the national land control planning, strengthening the use control of national land space and strictly protecting high-value carbon sink spaces. At the same time, we should promote ecological restoration in the national territory and increase carbon sinks in ecosystems.

Second, urban development needs to respect nature and make good use of planning for top-level design. Among them, transport distance is the concentrated expression of the basic spatial characteristics of human and nature, as well as the interaction between humans and space. In particular, the geographical distance, density, segmentation, and heterogeneity have great influence on the carbon emission of cities. For example, Russia is much larger than Mexico, with a land area of 8.7 times greater. As a result, although Russia and Mexico have the same population and per capita GDP in 2021, Russia’s per capita carbon emission of 12.04 tons is 3.2 times that of Mexico. According to the “Research on the Impact of Land Use Structure on Air Pollutants and carbon Emissions” project, Chinese urban and construction land accounts for 1% of the country’s total land area, but carbon emissions account for nearly 90% of the country’s total emissions. In addition, if the construction land scale doubled, the carbon emissions will increase by 1.7 times. Therefore, we need to conduct scale constraint and structural adjustment for all kinds of land development and construction based on geographical distance, density, segmentation, and heterogeneity. At the same time, we need to establish natural ethics, caring for the land, and guide residents to transform their behavior to the green and low-carbon direction.

Finally, ecology is a resource that can be transformed into assets, and its land factors allocation needs the assistance of the market. Through the market mechanism, ecological function can be transformed into ecological values and the modernization of harmonious coexistence between man and nature can be realized. Ecological environment and its functional diversity determine the different attributes of ecological value. Some ecological values take the products and services derived from the good ecological environment to meet certain needs of people as the carrier, such as ecological agricultural products, ecological tourism, etc., which are directly produced for peoples’ consumption or use, so as to obtain certain monetary benefits and realize value transformation. In practice, if the economic value of natural resources cannot be fully tapped, the ideal of “lucid waters and lush mountains are invaluable assets” will not be realized. If we cannot benefit from ecological protection and ensure the simultaneous development of social benefits and corresponding economic benefits, ecological builders will lose their enthusiasm for ecological protection and construction, and ecological environmental construction will lose its power source. As far as we are concerned, ecological products and ecological industrialization are an important means to realize the ideal of “lucid waters and lush mountains are invaluable assets”.

### 4.4. Interaction of Production-Living-Ecological Spaces: A Perspective from Livable City

**Figure 2:**
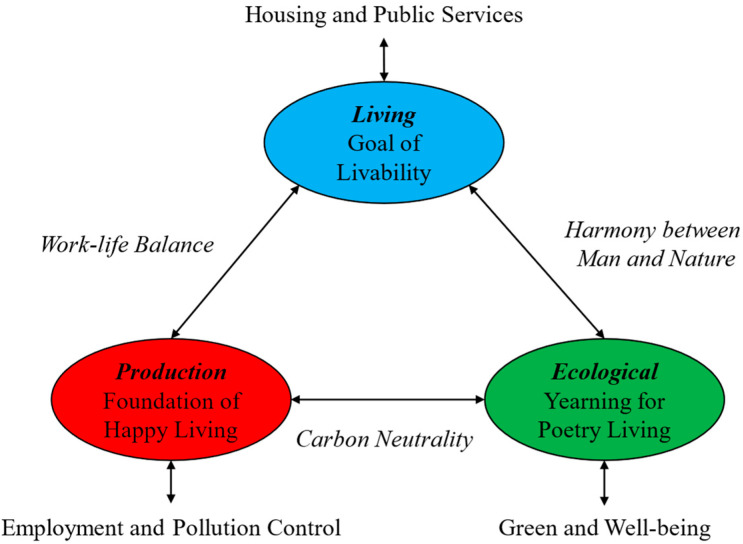
The interactive relationship of production–living–ecological spaces.

The modernization of China’s territorial space governance system and capacity looking forward to 2035 not only requires production, living, and ecological spaces to achieve their development goals respectively, but also requires an overall insight into the interactive relationship among production–living–ecological spaces to make allocation of land factors better serving for the optimization of territorial space layout at different scales. Cities are the crystallization of modern human civilization. Human yearning for “livable cities” has been reflected in Howard’s “Garden City” concept as early as the end of the 19th century. In 1933, The Athens Charter, the representative of urban spatial planning theory, put forward the overall concept of the coordinated development of the city and its surrounding areas, and divided the urban functions into work, residence, and recreation. This classification perspective coincides with production–living–ecological spaces, where the work function corresponds to production space, the residential function corresponds to living space, and the recreational function corresponds to ecological space. China’s development plan for 2035 also regards the construction of the “livable city” as an important goal. Therefore, we try to take the construction of the “livable city” as an example to specifically explain the interactive relationship between production–living–ecological spaces (Figure 2).

Livability is the primary goal of building a “livable city”. In urban spaces, the need for intimate, continuous relationship comes first. Therefore, urban living spaces need to pay close attention to the basic needs of people including social and spiritual needs. This requires that urban planning must be primarily carried out at the human scale to provide the necessary housing and basic public services. Second, production and living are closely related, and the construction of a modern livable city cannot be separated from the drive of production, which provides the necessary material basis for human beings to live happily. On the one hand, innovative behavior in urban production space will continue to provide employment for its residents, but on the other hand, it is also necessary to control the pollution caused by agglomeration in production space. Furthermore, a livable city should try to achieve a work-life balance as much as possible, ensure that wages and rents match, and ensure that green spaces and fresh air are not sacrificed to earn wages [43]. Finally, the urban ecological space reflects humans’ longing for poetic living. A beautiful ecological environment can not only directly promote human physical and mental health and realize “harmony between man and nature”, but also provide nature-based habitat through ecological resource value conversion. In addition, ecological space can also promote the sustainable development of production space through carbon neutrality. As a result, production–living–ecological spaces of a livable city have achieved effective interaction and established a virtuous circle. The overall optimization of livable urban space that integrates production–living–ecological needs to systematically formulate land use policies, fully consider the interactive relationship between production-living-ecological spaces in the allocation of land factors. The integration of “truth-kindness-aesthetic” in urban space is crucial to improving the overall welfare of the city and achieving sustainable urban development.

## 5. Findings

### 5.1. The Logics of Planning and Market Allocation of Land Factors

In the face of an increasingly complex global environment, the allocation of land factors shoulders the important task of coordinating development and security in the modernization of a country. Planning and market essentially represent two different logics of land factors allocation. The control and guidance functions of planning help build a security barrier for the development of national modernization, reflecting the “top-down” factors allocation logic, which is a manifestation of the modernization of the national governance system. The incentive and adjustment functions of market are the fundamental driving force to promote the country’s development to a new level, reflecting the “bottom-up” factors allocation logic, which reflects the modernization of national governance capacity. On the one hand, the modernization of China’s territorial space governance system and governance capacity, needs to follow the “top-down” logic, which starts from the continuity of the planning system and the overall coordination of the region, and realizes the allocation of land factors based on maximizing the overall social benefits. On the other hand, it also needs to follow the “bottom-up” land factors allocation logic to meet the interests of different individuals and stimulate the vitality and efficiency of market mechanism. Therefore, to realize the complementarity of public interests and personal interests in the development of national modernization requires a good connection between “top-down” and “bottom-up” logic in terms of allocation of land factors.

### 5.2. The Theoretical Mechanisms of Market and Planning Allocation of Land Factors

**Figure 3:**
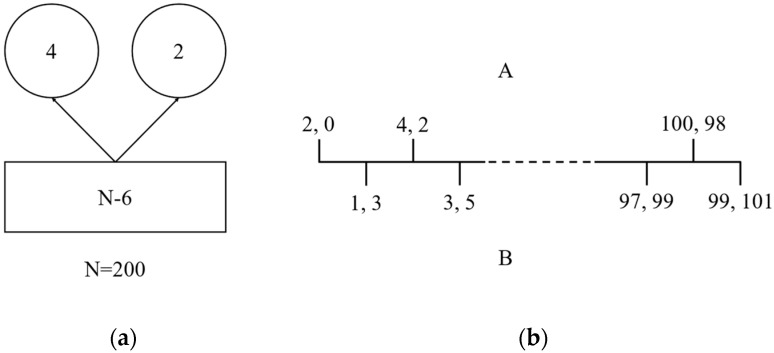
Centipede game model: (**a**) Description of game item: a square box with N − 6 gold coins and two round containers with 4 and 2 gold coins respectively; (**b**) Description of the game process. Before the comma is the return of A, after the comma is the return of B.

Individual rationality is the starting point of market allocations of land factors. To explain this argument, we introduce the “Centipede Paradox” model (Figure 3). It is a kind of paradox found in the study of game theory and game logic, and it is a kind of paradox of reasonable behavior choice. This game is called the “centipede game” because it spreads like a centipede. It means that two players have a square box with N gold coins and two round empty containers. First, you take two gold coins out of the box and put them both in one of the containers. Then every time after that, you take two gold coins out of the box and put one gold coin in each of the containers. The two players, A and B, take turns choosing strategies to either end the game, choose the container with the most coins, or let the game continue. Suppose A chooses first, then B, then A, and so on, and the number of games between A and B is a finite 100 times. The respective returns of this game are shown in Figure 1. For the first time, if A completes the decision, A and B get 2 and 0 gold coins, respectively. For the second time, if B’s decision ends, A and B get 1 and 3 gold coins, respectively. For the third time, if A completes the decision, A and B get 4 and 2 gold coins, respectively, and so on. Based on the logic of the game, the rational person’s assumption is that A is going to end the game on the second to last step. But the problem is that B is also smart, he anticipates A’s motivation, and he ends the game on the third to last move. It is not hard to see that in the reasoning process of this game, backward induction is used. If the market pursues individual rationality too much, the composite whole may be irrational. Therefore, planning is needed to supplement and correct market failures in the allocation of land factors.

**Figure 4:**
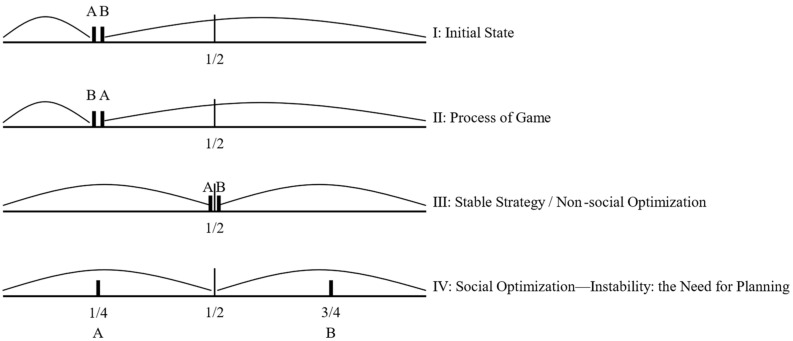
Social optimization model: A and B are two ice cream stands on the coastline that offer exactly the same goods and services.

Overall rationality is the starting point of planning allocations of land factors. A social optimization model is introduced here to illustrate this point [44]. As shown in Figure 4, there are two ice cream stands A and B on the coastline that offer exactly the same goods and services. In order to gain a larger market, the positions of A and B spontaneously developed from the initial state I to the stable state III of the balanced match during the game. To reduce the overall transportation cost of residents along the coast, the layout of ice cream stand A and B should be planned for social optimization, and A and B should be placed in 1/4 and 3/4 places respectively. However, overall rationality also needs to incorporate individual rationality. Facing the ever-changing economic and social development environment, over-emphasis on the control and constraints of planning may be counterproductive. The basic conclusion is that light control is the most effective, while tight control can lead to overreaction and sometimes even the disintegration of the machine. The compilation of territorial space planning needs to consider its variability, adjustability, selectivity and reconfigurability.

### 5.3. Enlightenment of Middle-Around Theory

In China’s reform to promote the modernization of territorial space governance system and capacity, we suggest that “middle-around” theory is a possible theoretical solution to effectively connect planning and market in the allocation of land factors. The western planning concept originates from city-state governance, with the city as the core and the bottom-up orientation as the starting point and the leading; however, the planning concept of China originated from irrigated agriculture. Since Yu controlled the flood, the core was rural areas, emphasizing top-down. In modern China, the influence of “Western learning to the east” and bottom-up Western planning concept influenced China. Especially after the reform and development in 1978, the western planning trend of thought had more and more profound influence on China. However, it conflicts with the deep-rooted top-down planning concept in China, so different plans have different ideas. It is under this background that “multi-planning integration” is proposed, and middle-around provides a new planning theory for the future “multi-planning integration”.

**Figure 5:**
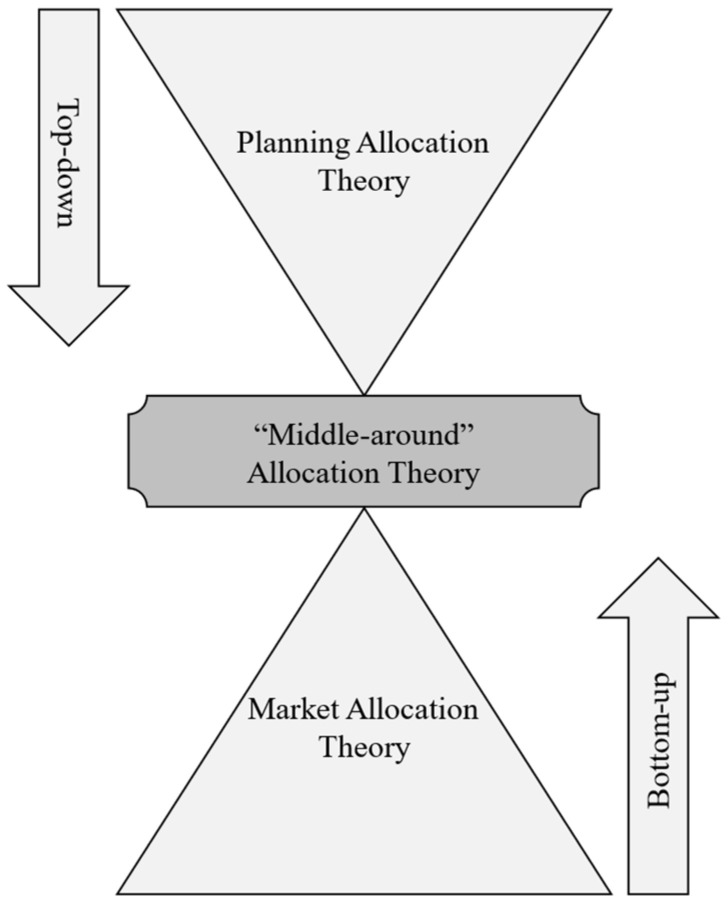
“Middle-around” theory in allocation of land factors.

Planning emphasizes goal orientation and usually starts from supply, so indicators are decomposed layer by layer from top to bottom. Market focuses on problem solving and tends to start from demand, which is a bottom-up demand orientation. “Middle-around”, also known as theory of “Waist”, is the intersection of “top-down” planning allocation theory and “bottom-up” market allocation theory (Figure 5), which is oriented by the balance between supply and demand, to achieve the synergy of achieving goals and solving problems. On the one hand, “middle-around” theory emphasizes that the rational allocation of land factors requires planning and market to be used together, neither cannot be neglected. On the other hand, it emphasizes that only by adaptively using government and market functions in face of specific problems in the allocation of land factors can avoid the logical conflict between “top-down” and “bottom-up”, and truly release the power of territorial space governance systems and capacity when driving modernization reforms.

From the perspective of production-living-ecological spaces, this study analyzes how China’s land factors allocation in the modernization development towards 2035 should use the two methods of planning and market to achieve the overall requirement of “intensive and efficient production space, livable and moderate living space, and beautiful ecological space”. Territorial space is extremely complex, but spatial scale is an entry point to understand the internal rules of it. Combining the guidance of “middle-around” theory and the starting point of spatial scales heterogeneity, we can better understand the differential mode of government-market collaborative allocation of land factors of production–living–ecological spaces. The first is global/country scale, which considers whether land resources can be used. In the case of available land, it can be divided into use/non-use. The theory needed here is sustainable development theory, including land ethics, global climate change and suitability assessment; the second is regional scale, considering the scale allocation of agricultural land and the utilization of non-agricultural construction land. The methods used here are demand forecasting and indicator decomposition. Demand forecasts must take into account the diversity of needs, including sustenance of food and housing, as well as agricultural and industrial productivity. Index decomposition involves structural adjustment; the third is local scale, considering the spatial layout, specifically the relationship between ecological land and construction land, as well as the internal relationship of construction land. The spatial layout should consider the social development stage, the influence of utilization and zoning planning, land use, location and transportation and other factors. To sum up, “middle-around” theory can provide helpful solutions to the practice of land factors allocation in developing countries, but the perfection of the theoretical solution always needs practice tests, timely feedback, and continuous improvement.

## 6. Conclusions

Planning and market are two means to allocate land factors, but there is a boundary between them. The territorial space is a complex system, current knowledge and cognition of human beings is limited, and the future is full of numerous variables and uncertainties. In this context, land factors allocation requires the synergistic allocation of planning and market to achieve both development and safety goals looking forward to 2035. Furthermore, land factors allocation should also consider the spatial scale differences [45]. To be specific, the adoptions to planning and market should follow the rule of “globally fuzzy to locally accurate” from top level to down level. The higher-level planning should be more macroscopic, more standardized, and more stylized. When it comes to detailed planning at lower level, the expression of spatial elements is more refined. For large-scale planning, it is necessary to do a good job of strategic guidance, coordinate the development goals and the bottom line of safety. For planning elements that must be implemented, such as urban development boundaries, permanent basic farmland red lines, and ecological protection red lines, planning should be strictly formulated, and relevant laws and regulations should be implemented. However, Technical standards can be in the form of guidelines, recommendations, etc., to provide flexible solutions to uncertainties, and leave enough room for market. For small-scale planning, due to the basic data information of clear research on market demand, the planning can be made clearer and more detailed. The layout of various spaces and facilities shall be coordinated to fully reflect the regional and cultural characteristics according to the local population and resource conditions, the stage of economic and social development, and the improvement requirements of the human settlements. Besides, it needs pay attention to the dynamic monitoring in the later period, so that the planning can be effectively implemented in the long run.

## Figures and Tables

**Table 1:** The proportion of city size in different development types of countries.

Size of Population	Low-Income Countries (%)	Middle-Income Countries (%)	High-Income Countries (%)
Small settlements: Under 20,000	73	55	22
Middle Settlement: 2 million to 1 million	16	25	26
The Big Settlement: More than 1 million	11	20	52

Source: World Development Report 2009.

## Data Availability

Data in Table 1 is originally cited from *World Development Report 2009: Reshaping Economic Geography*, p. 61 Table 1. “The size of urban settlements grows with development”.
